# Physiological expression of lens α-, β-, and γ-crystallins in murine and human corneas

**Published:** 2010-12-15

**Authors:** Shengwei Ren, Ting Liu, Changkai Jia, Xia Qi, Yiqiang Wang

**Affiliations:** Shandong Provincial Key Lab of Ophthalmology, Shandong Eye Institute, Shandong Academy of Medical Sciences, Qingdao, China

## Abstract

**Purpose:**

How corneal transparency is formed/maintained remains largely unclear. A group of enzymes which are referred to as enzymatic crystallins were proposed to contribute to corneal transparency in various animals. This study investigated whether the three classical lens crystallins, namely α-, β-, and γ-crystallins, exist in mouse and human corneas.

**Methods:**

Mice, human tissues, and cultured corneal cells were studied. The expression of lens crystallins in corneas or in cultured corneal cells were detected at the mRNA level by quantitative reverse transcription-PCR (QRT–PCR) and at the protein level by immunohistochemistry or western blotting. To check the effect of exogenous factor on expression of lens crystallins, cultured corneal cells were challenged with lipopolysaccharide or hydrogen peroxide and the expression of lens crystallins was monitored.

**Results:**

QRT–PCR revealed that the relative expression level of lens crystallins in C57BL/6 corneas were higher than in Balb/c corneas. Immunohistochemistry study showed that expression of αA-crystallin started from the embryonic stage, lasted untill old age, and was largely restricted to the epithelium or endothelium of the corneas. β- and γ-crystallins also were found in murine corneal epithelium. Upon treatment with lipopolysaccharide or hydrogen peroxide of cultured corneal epithelial cells, lens crystallins expression was significantly increased as detected by QRT–PCR or western blot assay. Further, both fetal corneal epithelial cultures and limbal stem cell cultures from adult human tissues were positive for lens crystallin immunofluorescence or immunohistochemistry staining.

**Conclusions:**

Lens crystallins are expressed in mammalian corneas and cultured corneal cells. The expression levels depended on the animal strains or cell status. The physiologic and pathological significance of lens crystallins in corneas deserves more investigation.

## Introduction

Compared to our understanding of lens transparency, much less is known about the mechanisms underlying the maintenance or loss of corneal transparency. It is tempting to apply theories and methodologies employed in studies of lens to those of cornea, since they not only share a similar morphogenesis but also are functionally related. Data accumulated in recent years showed that various corneal crystallins, which are also referred to as enzymatic crystallins due to their enzyme characteristics, were important contributors to the maintenance of corneal transparency in different species [1-3]. In such aspect, corneas utilize these proteins as both structural constituents and metabolic modulators to maintain their transparency [4,5]. Well defined examples of corneal crystallins include glutathione-S-transferase (GST)/Ω-crystallin [6], aldehyde dehydrogenase (ALDH) 3Aβ [7], ALDH1A1/η-crystallin [3,8], α-enolase/τ-crystallin [6,9], arginino-succinate lyase/δ-crystalllin [6], lactate dehydrogenase (LDH)/ε-crystallin [10], transketolase (TKT) [11], and gelsolin [12]. The list continues to grow, to which GAPDH/π-crystallin [13], triose phosphate isomerase (Tpi) [9], and scinderin-like gene [14] were added in recent years.

Prompted by the fact that enzymatic/corneal crystallins exist in both corneas and lenses, scientists assume these two neighboring and functionally related mini-organs jointly form a unit called “refracton” by sharing similar mechanisms for maintaining or losing transparency [2,15,16]. While corneal crystallins are readily detected in corneas of various animals [6,7,17,18], and the classical lens crystallins (i.e., α-, β-, and γ-crystallins) are also detectable in corneas of toads and frogs [19], few studies addressed whether the lens crystallins exist in mammalian corneas. To make it worse, limited available information is controversial [20-22]. For example, Flugel [20] used immunohistochemistry to show that corneal endothelium, and not other parts of the cornea, stained positive for αB-crystallin in humans. But Reddy [21], using similar methods, found no expression of αA- nor αB-crystallin in human corneas, and Robinson [22] recorded that αB-crystallin mRNA was detected only in the endothelium and not in the epithelium at day 14 after birth of mice. In an effort to identify differential expressed gene during development of murine corneas using microarray method, Wu et al. [23] recently showed that several lens crystallin genes including alpha crystallin A (*Cryaa*), beta crystallin A1 (*Cryba1*), beta crystallin B2 (*Crybb2*), gamma crystallin B (*Crygb*), gamma crystallin C (*Crygc*), gamma crystallin D (*Crygd*), gamma crystallin F (*Crygf*) and gamma crystallin S (*Crygs*) were expressed in murine corneas (at the mRNA level) but did not make an effort to present any other proof. In a project concerning gene profiling in murine experimental corneal neovascularization models, we also found that normal murine corneas contained high amounts of lens crystallin mRNA (unpublished). Here, using a combination of methods, we demonstrated the physiologic expression of three main types of lens crystallins in both murine and human corneas.

## Methods

### Subjects

Mice, human tissues, and cultured corneal cells were used in this study. Specific-pathogen-free Balb/c and C57BL/6 mice were purchased from Beijing Pharmacology Institute, Chinese Academy of Medical Sciences (Beijing, China). Each cornea was inspected under a slit lamp microscope to exclude any corneas with abnormality. The ARVO Statement for the Use of Animals in Ophthalmic and Vision Research was observed throughout the study. A murine corneal epithelial cell line TKE2 cells, a gift of Dr. Kawakita at Keio University School of Medicine (Tokyo, Japan) [24] was also used. For studies with human tissues, all related protocols were approved by the Ethics Committee of Shandong Eye Institute and the tenets of Declaration of Helsinki were observed. Intact corneas were from miscarried fetus, and limbal rims were from donor corneas during the preparation of corneal grafts for keratoplasty. For preparation of primary epithelial cell cultures, an explant culture method [25] was used with slight modification. Briefly, the human corneal tissues were cut into pieces of about 2 mm in dimension and placed in culture dish with epithelial side downward for 15 min before 2 ml culture medium (1:1 mixture of Dulbecco’s modified Eagle’s medium and Ham’s F-12 medium supplemented with 10% fetal bovine serum, 5 mg/ml insulin, 0.1 nM cholera toxin, 10 ng/ml epidermal growth factor, and 50 IU/ml penicillin–streptomycin) per 35 mm dish was supplemented onto explants. Culture medium was replenished after 24 h and every other day from then on. The explants were removed one week later. It usually took two weeks for the culture to reach confluent (P0) for direct use or for passage (P1). All primary cultured cells were used before or at second passages (P2).

### Treatment of corneal epithelial cells with proinflammatory stimuli

TKE2 cells were grown on coverslips laid in 24 well plates in keratinocyte-SFM (Gibco, Grand Island, NY) and treated with 1 μg/mL lipopolysaccharide (LPS) or 200 μM hydrogen peroxide (H_2_O_2_) for 24 h (for quantitative reverse transcription-PCR [QRT–PCR] and western blot assay) or 4 days (for immunofluorescence assay). Further assays performed with these cells were described in details below.

### QRT–PCR

Total RNA was extracted from corneas or cultured cells using isopropanol precipitation and purified using NucleoSpin® RNA clean-up columns (MACHEREY-NAGEL, Düren, Germany). One microgram of total RNA from each pooled sample was reverse transcribed into cDNA using a PrimeScript RT Reagent Kit (Takara BIO INC., Shiga, Japan) according to the protocol provided by the manufacturer. QRT–PCR was performed using the Taqman method with proper primers and probes for interested genes (Table 1). Ribosomal protein L5 (*RPL5*) gene was used as the reference gene. Reactions for each sample were performed in triplicate using an ABI 7500 Detection System (Applied Biosystems, Foster City, CA) and a PCR protocol comprising an initial 10 min incubation at 95 °C followed by 40 cycles of 15 s at 95 °C and 1 min at 60 °C. The raw data were analyzed using SDS 7500 software (Applied Biosystems) and C_t_ values for each gene in each sample were determined for further analysis.

**Table 1:** Primers and probe sequences used for QRT–PCR.

**Gene**	**Forward primer**	**Reverse primer**	**Probe**
αA1-crystallin	TCTACCCCAGCCGACTGTTC	GGGCAGCAGGTCGTACTCAA	FAM-ACCAGTTCTTCGGCGAGGGC-TAMRA
βA1-crystallin	GGGCAAGAGGATGGAGTTCA	TGACCGGACATTATCAAAATTACG	FAM-AGCTCCTGCCCAAATGTCTCTGA-TAMRA
βB2-crystallin	AGAACTTCCAGGGCCATTCC	CTCCATACCAGTCTCCTTCAGGTT	FAM-ACGAGCTCAGCGGGCCCTG-TAMRA
RPL5	GGAAGCACATCATGGGTCAGA	TACGCATCTTCATCTTCCTCCATT	FAM-TGTGGCAGACTACATGCGCTACC-TAMRA

### Western blotting

All primary and secondary antibodies for western blotting and immunohistochemistry were purchased from Santa Cruz Biotechnology Inc., (Santa Cruz, CA) if not specified elsewhere. The corneas were obtained from six- to eight-week-old normal female C57BI/6 or Balb/c mice. Three corneas were pooled, respectively, as one sample. Protein was extracted from corneas or cultured cells using the RIPA lysis buffer (50 mm Tris PH7.4, 150 mm NaCl, 1%Triton X-100, 1% sodium deoxycholate, 1% SDS, sodium orthovanadate, and sodium fluoride; Beyotime, Shanghai, China) according to the manufacturer’s instructions. Each of the prepared samples in a final volume of 10 μl containing a total of 50 μg of protein was resolved on 15% SDS–PAGE gels for 1.5 h at 120 V and then transferred to nitrocellulose membranes (Millipore, Billerica, MA). The blots were blocked in 5% nonfat dry milk dissolved in TBST buffer for 1 h, incubated with monoclonal mouse anti-αA-crystallin (sc-28306) or anti-GAPDH (KC-5G5; KangChen Biotech, Shanghai, China) antibodies in TBST for 1 h, followed by incubation with HRP-conjugated goat anti-mouse IgG antibody (MAXIM BIO, Fuzhou, China) for 1 h. All incubations were done at room temperature, and three washes with 10 ml TBST buffer were applied between each step. The membranes were then developed with SuperSignal West Femto Maximum Sensitivity substrate (Pierce Biotechnology, Rockford, IL) and exposed to X-ray film (Kodak, Rochester, NY). The bands were analyzed using NIH Image 1.62 software (NIH, Bethesda, MD). For each sample, the levels of αA-crystallin were normalized to that of GAPDH.

### Histology and immunostaining

Murine eyeballs or human corneal tissues were embedded in a paraffin block and subjected to routine hematoxylin-eosin (HE) staining and immunohistochemistry. Monoclonal mouse anti-αA-crystallin in combination with horse-radish peroxidase (HRP)-conjugated goat anti-mouse IgG antibody (MAXIM BIO) was used. After developing with 3, 3′-diaminobenzidine, the sections were counterstained with hematoxylin. For immunofluorescence staining of the cultured corneal cells, the cells were fixed with acetone and stained with monoclonal mouse anti-αA-crystallin (sc-28306), polyclonal goat anti-αB-crystallin (sc-22391), polyclonal rabbit anti-β-crystallin (sc-22745), and polyclonal rabbit anti-γ-crystallin (sc-22746). Fluorochrome conjugated secondary antibodies included goat anti-mouse IgG-FITC (sc-2010), goat anti-mouse IgG-TR (sc-2781), donkey anti-goat IgG-FITC (sc-2024), goat anti-rabbit IgG-FITC (sc-2012), and goat anti-rabbit IgG-TR (sc-2780). The nuclei were stained with DAPI. All sections were observed using an E800 fluorescent microscope (Nikon, Tokyo, Japan) with appropriate filters.

## Results

### Lens crystallins in normal murine corneas

**Figure 1:**
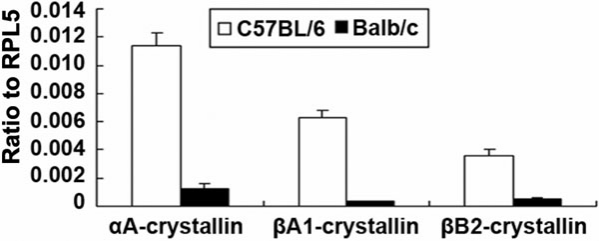
Expression of lens crystallins in murine corneas. C57BL/6, open; Balb/c mice, filled, both 6–8 weeks old. The relative intensities of genes versus RPL5 were obtained by comparison of amplification dynamic Ct for each gene in QRT–PCR assay. Each sample was run in triplicate wells in PCR reaction, and the error bars represented standard errors for three samples in each group.

**Figure 2:**
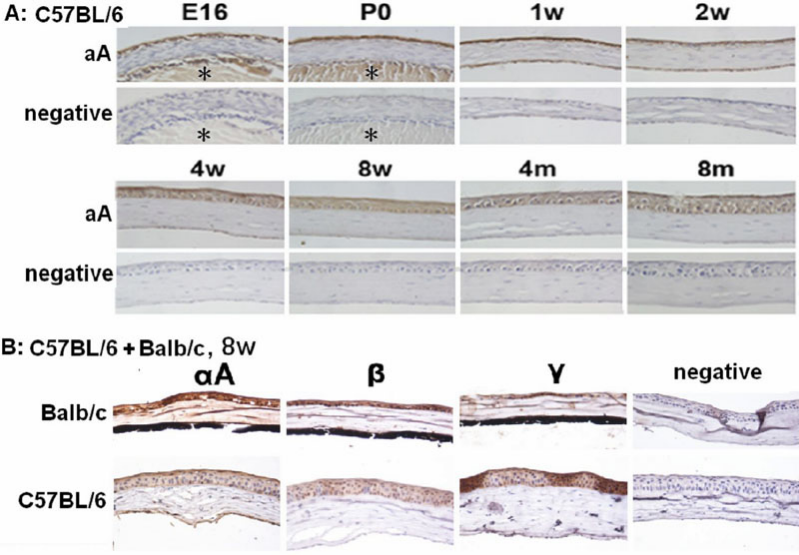
Lens crystallins expression in murine corneas of various ages and strains. Positive immunoreactivity is denoted by a brown color. **A**: Alpha-A expression in C57BL/6 corneas at different development ages. E16, embryonic day 16; P0, newly born; w, weeks; m, months. Each sample was serially sectioned and the ones near the corneal centers were used for immunohistochemistry. Neighboring two sections were processed exactly same except for that the primary antibody was omitted for one of them and used as staining negative control (lower rows). Asterisks (*) in E16 and P0 samples denote tissue of lens. **B**: Expression of lens crystallins in murine corneal epithelium of C57BL/6 or Balb/c strains.

QRT–PCR revealed that the relative expression level of lens crystallins in corneas were different in normal adult Balb/c and C57BL/6 mice (Figure 1). In most of the following experiments only αA-crystallin was focused on as a representative. Using corneas form C57BL/6 mice of different ages, it could be shown that expression of αA-crystallin in the corneas started from embryonic stage (embryonic 16 days) and lasted till old age (8 months in this study). Throughout this period, αA-crystallin in the epithelial layers remained at a relatively high and stable level, but its expression in corneal endothelium was strong at early life stage (e.g., 1 week) and gradually diminished at old age (e.g., 8 months), while its expression in stromal layers was always marginal (Figure 2A). Comparison to the co-stained embryonic lens (E16 and P0) manifested that the αA-crystallin protein level in corneal epithelium could be even higher than in lens at this stage (Figure 2A). Similarly, β- and γ-crystallins expression in corneas was confirmed with both strains of mice at 2 months of age (Figure 2B).

### Lens crystallin expression in corneal epithelial cells responded to exogenous stimuli

**Figure 3:**
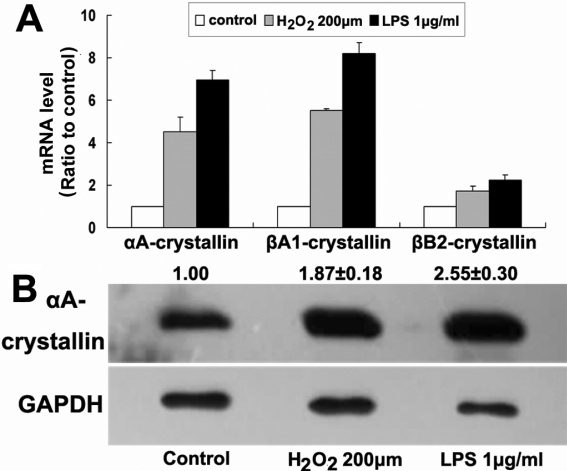
Lens crystallins expression responded to stimuli like H_2_O_2_ and LPS. **A**: Relative intensity of each gene to RPL5 detected by QRT–PCR in untreated cells (control) was arbitrarily set as 1.0. Shown was one representative of three similar experiments. **B**: In western blot assay, ratio of αA-crystallin intensity to that of GAPDH in control cells was arbitrarily set as 1. Numbers above the αA-crystallin bands (mean±standard error) were obtained from three independent experiments. respectively.

To check whether lens crystallins are functionally involved in the physiologic response of corneal cells to exogenous stimuli, we used H_2_O_2_ and LPS as oxidative and inflammatory stimulus, respectively, and studied their potential effects on expression of lens crystallins in murine corneal epithelial cells. Upregulation of αA- and β-crystallin in response to H_2_O_2_ and LPS treatment were detectable in TKE2 by using QRT–PCR assay (Figure 3A). In representative experiments, upregulation of αA-crystallin in response to H_2_O_2_ and LPS was further confirmed by using western blot assays (Figure 3B).

### Lens crystallins were expressed in human corneal cell cultures and human corneas

**Figure 4:**
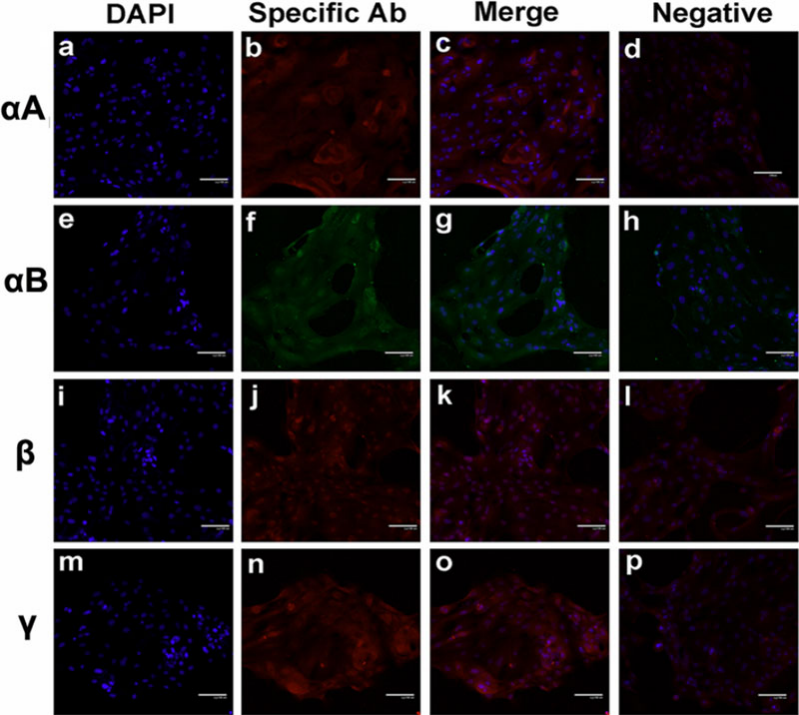
Lens crystallins expression in primary culture of human fetal corneal epithelial cell culture. Intact corneas from miscarried fetus were used for culture of primary corneal epithelial cells, and second passages were used for immunofluorescence staining for the proteins of αA-,αB-,β- and γ-crystallin using different combination of primary and fluorescence conjugated secondary antibodies. The nuclei were stained with DAPI. Each section was observed under appropriate filters and the image of nuclei was overlaid on image of protein staining (“merge”), respectively. For background controlling, the primary antibodies were omitted in parallel protocol but only stained with secondary antibodies (“negative”).

**Figure 5:**
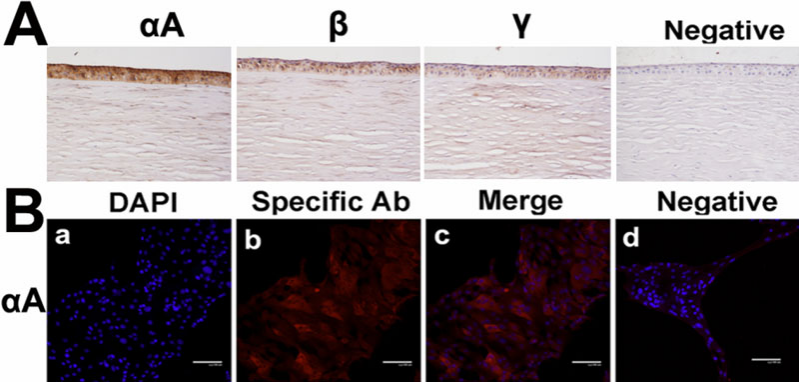
Expression of lens crystallins in corneal limbal rim of adult human donor or cultured corneal epithelial cells. The limbal rims were obtained during preparation of corneal grafts for keratoplasty. **A**: Routine immunohistochemistry protocol was performed using proper combination of proper anti-crystallin primary and HRP-conjugated secondary antibodies. The sections were developed with 3, 3′-diaminobenzidine and counterstained with hematoxylin. B: Part of the limbal rims was used for culture of corneal epithelial cells and stained as described in the Figure 4 legend.

We next extended our studies to the human system. Both fetal corneal epithelial cultures and adult limbal stem cell cultures were positive for lens crystallin immunofluorescence staining, implying that human corneal epithelial cells express lens crystallins effectively (Figure 4 and Figure 5). The peripheral rings left from normal donor corneas during graft preparation were also positive for αA-, β-, and γ-crystallin staining (Figure 5).

## Discussion

To the best of our knowledge, this is the first systematic study to show that all three main lens crystallins, besides the well documented corneal crystallins, exist in mammalian corneas. The main findings of the current study include: (1) lens crystallins expression in murine corneas starts at early stage of embryonic development and might last for whole life; (2) lens crystallins expression levels in certain strain of mice depend on its genetic background; (3) lens crystallins expression in corneas under physiologic status might be a universal phenomenon among mammals; (4) lens crystallin expression is responsive to various stimuli such as LPS and H_2_O_2_. Though these findings imply that lens crystallins are among the main constituents of transparent corneas under physiologic conditions, and it might be tempting to take a step further to assume that these proteins might play important roles in corneal transparency, other existing evidence seems to argue against such assumption. For example, the many mutations of lens crystallins that cause congenital cataracts are rarely found to cause corneal transparency abnormalities, even though such congenital cataracts are often accompanied by microcornea symptoms [26-30]. Further more, targeted disruption of the α-crystallin genes in mice caused cataracts, but the corneas of these α-crystallin-deficient mice looked normal [31]. In fact, similar apparently controversial observations had been recorded for corneal crystallins. In brief, corneal crystallins like ALDH1 and ALDH3 are believed to play important roles in maintaining the proper state and function of corneas in various species [2,3], but not any abnormalities had been found in the corneas of ALDH3a1- or ALDH1a1-deficient mice [32,33], suggesting either that corneal crystallins in corneas are less important than previously proposed or that mutation-caused dysfunction of corneal crystallins could be properly compensated in corneas due to redundancy and functional overlapping of various lens crystallins. We propose that this principle or hypothesis applies to lens crystallins in corneas too. Thus, more strictly controlled clinical studies using subjects known to host various mutations in lens crystallin genes, or experimental studies of the biologic properties of corneal cells from crystallin-deficient mice, will help to define whether these physiologically expressed lens crystallins in corneas play any important role in transparency or other bioprocess of corneas. This said, the studies about the lens crystallins in other sites rather than lens or in other pathological processes beside cataract [34,35] should provide reference for such exploration. For example, it has been known that the retina used α-, β-, and γ-crystallins as one mechanism to protect against infection- or oxidative stress-induced tissue damage [36-39]. More recently, it was found that certain population of retinal pigment epithelial cells in retina from age-related macular degeneration patients undergo change to express αB-crystallin [40]. Kase et al. [41], using a choroidal neovascularization model, further showed that αB-crystallin might act as a chaperone for VEGF in angiogenesis. Earlier studies by Dimberg et al. [42] also showed that αB-crystallin promotes angiogenesis in tumor development. Thus it might be helpful if the potential role of lens crystallins in pathological process like corneal neovascularization or infectious keratitis be explored.

As shown in the immunohistology study, αA-crystalllin level in corneal epithelium was comparable to that in developing lens (Figure 2). This might lead to suspicion why few previous studies detected lens crystallins in mammalian corneas in various gene banks related to the corneas, just like in the studies deposited in the NEIBank [43]. Without a conclusive answer to this question, we proposed that the sensitivity or accuracy of investigative methods used in different studies determined whether lens crystallins were detectable. To check our hypothesis, we reviewed the expression pattern of a couple of lens crystallin genes in several public murine microarray data sets in the Pubmed Gene Expression Omnibus (GEO) [44]. Data set GSE11900 compared the gene expression patterns in corneas of postnatal day 10 and 7-week old mice [23]. In the adult group, the average MAS5 normalized values of signal intensities for transketolase (*Tkt*) (16173.1), *Cryaa* (6353.6), *Cryab* (395.3), *Cryba1* (3758.2), *Crygb* (1810.9), and reference genes glyceraldehyde 3-phosphate dehydrogenase (*Gapdh*) (4836.9) and beta-actin (*Actb*) (10801.5) should be interpreted as that *Cryaa*, *Cryba1*, *Crygb*, and the corneal crystallin gene *Tkt* were expressed abundantly in adult corneas, but *Cryab* was expressed at much lower level. In another study producing GSE14270, however, Lively et al. [45] compared the gene expression patterns of corneas from three mouse strains that show different central corneal thickness. According to that data set which was normalized with Guanine Cytosine Robust Multi-Array (GC-RMA) analysis, the average values of signal intensities for the same batch genes in adult C57BL/6J mice were 19815 0.1 (*Tkt*), 4.2 (*Cryaa*), 6565.6 (*Cryab*), 4.6 (*Cryba1*), 5.2 (*Crygb*), 14105.5 (*Gapdh*), and 18977.2 (*Actb*), implying that Cryab was highly expressed but other several lens crystallin genes did not express at all. Similar inconsistency was observed with other lens crystallin genes or in other study systems (data not shown). Thus more extensive experimental studies or data mining are necessary to alert scientists of the neglected lens crystallins in the corneas.

In summary, we report here that lens crystallins are abundantly expressed in mammalian corneas, and their expression levels change under exogenous stimuli that might be related to imflammation and infection. We propose that lens crystallins play a role in physiologic functions of corneas and might also serve as foundation for the concept of “refracton.” Future studies of the functions of these crystallins in corneas will help to uncover the mystery of corneal transparency.
